# Development of a Low-Molecular-Weight Filtrate Reducer with High-Temperature Resistance for Drilling Fluid Gel System

**DOI:** 10.3390/gels9100805

**Published:** 2023-10-07

**Authors:** Fengbao Liu, Jinsheng Sun, Xianbin Huang, Yuan Geng

**Affiliations:** 1 National Key Laboratory of Deep Oil and Gas, China University of Petroleum (East China), Qingdao 266580, China20170092@upc.edu.cn (X.H.); 2 School of Petroleum Engineering, China University of Petroleum (East China), Qingdao 266580, China; 3 PetroChina Tarim Oilfield Company, Korla 841000, China; 4 China Petroleum Engineering Technology Research Institute Co., Ltd., Beijing 102200, China

**Keywords:** low molecular weight, high-temperature resistance, salinity tolerance, filtrate reducer

## Abstract

Currently, conventional polymeric filtrate reducers with high-temperature resistance for use in drilling fluids have high molecular weights, which greatly affects the rheological properties. Therefore, to address the challenges in regulating the rheology and filtration performance of high-density drilling fluids at high temperatures, it is essential to develop low-molecular-weight filtrate reducers with high-temperature resistance. In this study, a low-molecular-weight filtrate reducer with high-temperature resistance (LMF) was prepared via free radical polymerization from acrylamide and 2-acrylamido-2-methyl-1-propanesulfonic acid as monomers, tertiary dodecyl mercaptan as a chain transfer agent, and ammonium persulfate as the initiator. LMF was then characterized by infrared spectroscopy, thermogravimetric analysis, and gel permeation chromatography. The obtained filtrate reducer exhibits a weight-average molecular weight (*M*_w_) of 3819 and an initial thermal decomposition temperature of 300.7 °C, indicating good thermal stability. The effects of LMF dosage, temperature, and NaCl dosage on the rheology and filtration performance of mud samples were also investigated, and the mechanism of action was revealed by zeta potential, particle size distribution, scanning electron microscopy, and adsorption measurements. The results reveal that LMF increases the mud sample viscosity and reduces its filtration. For example, the filtration of the mud sample with 2 wt% LMF was 7.2 mL, a reduction of 70% compared to that of a blank mud sample. Further, after aging at 210 °C for 16 h, the filtration of the same sample was 11.6 mL, and that of a mud sample with 2 wt% LMF and 35 wt% NaCl after aging at 180 °C for 16 h was 22 mL. Overall, we have reported a scheme to prepare a low-molecular-weight filtrate reducer with high-temperature resistance and superior filtrate-reducing effects, laying the foundation for the investigation and development of low-molecular-weight filtrate reducers.

## 1. Introduction

Water-based drilling fluids have been increasingly adopted in oil and gas drilling because of their low cost and environmental impact [1,2,3,4]. Water-based drilling fluids comprise water, bentonite, and other functional additives [5,6,7]. Drilling fluid is a general term for circulating fluids that meet the needs of drilling work with various functions during the drilling process. The drilling fluid passes through the high-pressure manifold on the ground and reaches the drill bit through the drill pipe. During use, the drilling fluid circulates and flows in the annulus between the wellbore wall and the drill pipe, spraying out from the drill bit to clean the bottom of the well and carrying rock cuttings out of the well. The filtration performance of drilling fluid has an important impact on reservoir protection and wellbore stability. Chemical agents that can reduce the filtration amount of drilling fluid are called drilling fluid loss-reducing agents. Filtrate reducers are one of the most essential additives [8,9,10]. During the circulation of drilling fluids, because of the differential formation pressure, the fluid in the wellbore invades the geological formation and forms filter cakes on the wellbore surfaces, reducing wellbore stability and inducing downhole accidents [11,12,13]. Therefore, high-performance filtrate reducers for use in water-based drilling fluids that retain good fluid performance and ensure safety in drilling operations are urgently required.

For example, Li et al. [9] prepared a zwitterionic copolymer (ZCP) with strong adsorption on bentonite via hydrogen bonds and electrostatic force. This allowed the formation of thin and dense filter cakes, thus reducing filtration. Specifically, the filtration of drilling fluids with 2 wt% ZCP in saturated saline water after aging at 200 °C was only 9.6 mL. In addition, Zhu et al. [14] synthesized a modified xanthan gum derivative (XG-*g*-AAA) by grafting xanthan gum with acrylamide (AA), 2-acrylamido-2-methyl-1-propanesulfonic acid) (AM), and 2-Acrylamido-2-methyl-1-propanesulfonic acid (AMPS), and this showed superior temperature resistance and introduced shear thinning behavior in a water-based drilling fluid. Further, between 150 and 200 °C, the filtration of the drilling fluid with 3 wt% XG-g-AAA was less than 10 mL.

Zhang et al. [15] synthesized a filter loss-reducing agent ASML with hydrophobic association properties, which has a temperature resistance of up to 200 °C. Filtrate-reducing agents form a network structure in drilling fluid via the hydrophobic association of molecules themselves, significantly increasing the viscosity of the drilling fluid and reducing the filtration rate at high temperatures. At 200 °C, the filtration rate of drilling fluid containing 30 wt% NaCl is only 5 mL. Li et al. [16] prepared a new temperature and salt-resistant micro crosslinked amphoteric electrolyte gel DDAM, which was synthesized by free radical copolymerization of N,N-dimethylacrylamide, diallyldimethylammonium chloride, 2-acrylamido-2-methylpropane sulfonic acid, maleic anhydride, and chemical crosslinker triallylamine. At 200 °C, the apparent viscosity of drilling fluid containing 2 wt% DDAM is still as high as 28 mPa·s, with good filtration capacity. In addition, the application of nanomaterials [17] in the synthesis of filtration reducers has significantly improved the performance of drilling fluids. Dong et al. [18] synthesized a nanocomposite filtration reducer ANDP using modified nano lithium soap as raw material. When the dosage is 2.0 wt%, the viscosity of the base slurry can be increased by eight times, and the filtration loss of the freshwater base mud after aging at 200 °C is 10.4 mL. Moreover, Yang et al. [19] fabricated an organic–inorganic nanocomposite (P-RD’) containing laponite and polymers, which forms a 3D network structure in an aqueous solution and yields good rheological properties in water-based drilling fluids. After aging at 180 °C, the filtration of high-temperature, high-pressure (HTHP), and API American Petroleum Institute (API) drilling fluids drilling fluids containing 15 wt% NaCl were limited to 29.2 and 7.1 mL by P-RD’. Shan et al. [20] prepared a nanografted copolymer (EAANS) via inverse emulsion polymerization by grafting copolymers on the surface of nano-SiO_2_. After aging at 150 °C, the filtration of HTHP containing 2 wt% EAANS was only 21.8 mL.

Synthetic polymer fluid loss agents are usually water-soluble polymers. After the polymer fluid loss reducer is dissolved in the drilling fluid, the gel network structure is formed via the irregular molecular structure of the polymer, which significantly increases the viscosity of the drilling fluid to reduce the fluid loss. Therefore, the main mechanism of reducing the filtration rate by increasing the viscosity of drilling fluid is the use of filtration-reducing agents. The larger the molecular weight of water-soluble polymer filtrate reducer, the more able it is to maintain the viscosity of drilling fluid after high-temperature aging. The higher the viscosity of drilling fluid and the lower the filtration rate, the better its temperature resistance. However, drilling fluid requires the use of weighting agents to increase its density and balance formation pressure during use. The deeper the formation, the higher the temperature and pressure at the bottom of the well and the higher the density of the drilling fluid used. The density of drilling fluid increases, and its viscosity and shear force also sharply increase. Adding polymer fluid loss-reducing agents to regulate the filtration rate by increasing the viscosity of the drilling fluid will seriously affect the rheological properties of the drilling fluid and thus affect its application. Conventional polymeric filtrate reducers with high-temperature resistance usually have a high molecular weight, which can greatly increase the viscosity but impact the rheological properties of drilling fluids. In particular, for high-density fluids, a filtrate reducer that has little impact on the rheological properties is required to maintain the drilling fluid performance. Low molecular weight filtrate reducer does not significantly increase the viscosity of drilling fluid and has a good filtrate loss-reducing effect. Therefore, the development of low-molecular-weight filtrate reducers with high-temperature resistance is crucial. For example, Beg et al. [21] investigated the effects of low-molecular-weight poly(4-styrenesulfonic acid-*co*-maleic acid) sodium salt (PSSM) on the filtration performance of drilling fluids. They found that filtration was reduced by 63% and 75% in drilling fluid with 1 and 2 wt% PSSM, respectively. In addition, Dong et al. [22] synthesized a low-molecular-weight filtrate reducer with high-temperature resistance (LDMS) using *N,N*-dimethylacrylamide, sodium *p*-styrenesulfonate, and maleic anhydride. Mechanism studies have shown that low molecular weight filter loss-reducing agent LDMS can be inserted into the crystal layer of bentonite, enhancing the hydration performance of clay particles. Filtration tests revealed that, after aging at 210 °C, the filtration of the drilling fluid with 2 wt% LDMS was only 8.0 mL.

In this work, a low-molecular-weight filtrate reducer with high-temperature resistance (LMF) was prepared via free radical polymerization and was characterized using Fourier transform infrared spectroscopy (FTIR), thermogravimetric analysis (TGA), and gel permeation chromatography (GPC). The effects of LMF dosage on the filtration performance of mud samples were investigated, and the temperature resistance and salinity tolerance of LMF were also evaluated. Finally, the mechanism of action of LMF was revealed by zeta potential, particle size distribution, scanning electron microscopy (SEM), and adsorption measurements.

## 2. Results and Discussion

### 2.1. FTIR Analysis

**Figure 1:**
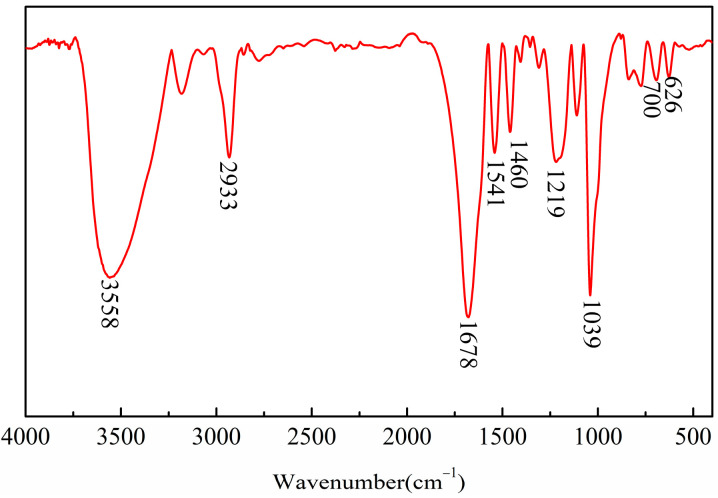
FTIR spectrum of LMF.

The FTIR spectrum of LMF (Figure 1) shows the presence of various functional groups that confirm the successful synthesis of the target product. Briefly, the absorption peak at 3558 cm^−1^ is caused by N-H tensile vibration. The absorption peak of 2933 cm^−1^ is caused by C-H vibration in -CH_3_. The absorption peak at 1678 cm^−1^ is a characteristic peak of C=O. The absorption peaks at 1541 cm^−1^ and 1460 cm^−1^ are caused by the benzene ring. The absorption peaks at 1219 cm^−1^, 1039 cm^−1^, and 626 cm^−1^ are caused by S=O, S-O, and O-S-O vibrations, respectively. The absorption peak at 700 cm^−1^ is caused by the bending vibration of C-H outside the benzene ring plane. The peak positions and their assignments are based on previous literature [9,23,24]. These results indicate that all LMF monomers participated in the reaction, and the target produced had been synthesized.

### 2.2. TGA

**Figure 2:**
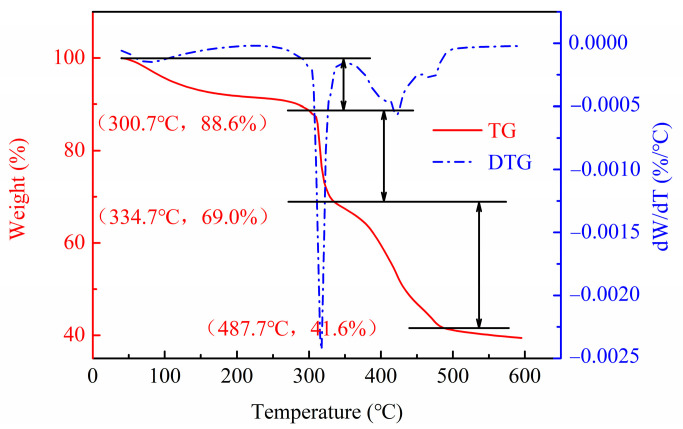
TGA curve of LMF.

The TGA curve of LMF (Figure 2) shows three stages of weight loss: (i) Before 300.7 °C, there was the first loss of weight, and at 300.7 °C, the mass residue rate was 88.6%, which was caused by the volatilization of free water and bound water in LMF [16,25,26]; (ii) between 300.7 °C and 334.7 °C, the filtrate reducer experienced a second loss of weight, with a mass residue rate of 69.0% at 334.7 °C. This is due to the molecular structure of the filtrate reducer beginning to undergo thermal degradation and (iii) at 487.7 °C, at which point LMF decomposition is completed. Thus, because decomposition only started at 300.7 °C, the LMF filtrate reducer has good thermal stability.

### 2.3. GPC Analysis

**Figure 3:**
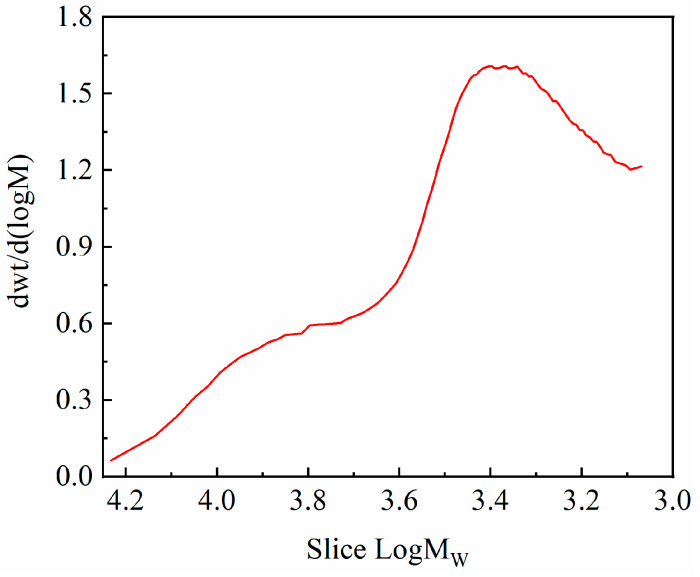
Analytical curve of LMF molecular weight.

Table 1 and Figure 3 showLMF’s relative molecular weight. As shown, LMF has a wide polydispersity index (*D* = 1.761093). Thus, the filtrate reducer is a copolymer with high polydispersity. LMF has a low weight-average molecular weight (*M_w_* = 3819). Low molecular weight polymers refer to those with a relative molecular weight between 1 × 10^3^ and 1 × 10^6^. The weighted average relative molecular weight of LMF is 3819, which meets the requirements of low molecular weight polymers for the relative molecular weight range, indicating that the synthesized filtrate reducer LMF is a low molecular weight polymer.

### 2.4. Evaluation of Rheology and Filtration Performance

**Figure 4:**
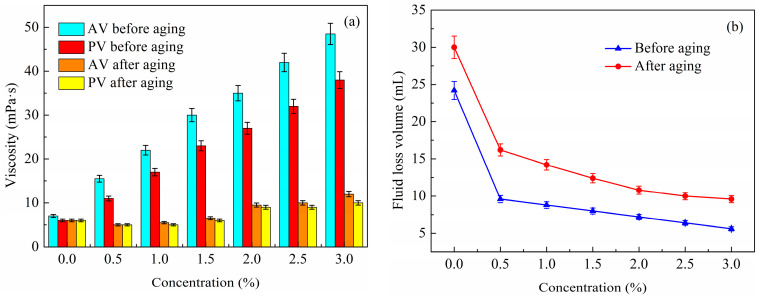
Effects of LMF dosage on the rheology (**a**) and filtration performance (**b**) of mud samples.

Figure 4a shows that the mud viscosity increases with LMF dosage but decreases after aging. However, despite the decrease in viscosity, it is still positively correlated with LMF dosage. LMF also reduced the mud filtration (Figure 4b). Specifically, the filtration of mud treated with 2 wt% LMF was 7.2 mL, 70% lower than that of the blank mud sample (24.2 mL). The apparent viscosity of the drilling fluid was 47 mPa·s, and the apparent viscosity of the base mud was 7 mPa·s, which has increased by nearly six times. The plastic viscosity of drilling fluid was 37 mPa·s, and the plastic viscosity of base mud was 6 mPa·s, which has increased by nearly five times. It can be seen that the viscosity-increasing effect of LMF before aging is good, and it has a good filtration loss-reducing effect. After aging at 180 °C for 16 h, the filtration of the same sample was 10.8 mL, 64% lower than that of the blank mud sample (30 mL). The apparent viscosity of the drilling fluid was 12 mPa·s, and the apparent viscosity of the base mud was 6 mPa·s, which has nearly doubled in viscosity. The plastic viscosity of the drilling fluid was 9 mPa·s, and the plastic viscosity of the base mud was 6 mPa·s. The plastic viscosity has increased by 50%. After the aging of drilling fluid, LMF has a weak effect on increasing the viscosity of the base mud, but it can also significantly reduce the filtration loss of the base mud. We all know that the viscosity-increasing effect of fluid loss-reducing agents is a very important mechanism in which they have a fluid loss-reducing effect [27,28,29,30]. From the test results, it can be seen that the filter loss-reducing agent LMF does not only reduce the filtration by increasing the viscosity of the drilling fluid. Thus, the results show that LMF has good filtrate-reducing capability, and the effect is linear. Further, the addition of LMF has little effect on the rheological properties of the drilling fluid.

### 2.5. Evaluation of Temperature Resistance

**Figure 5:**
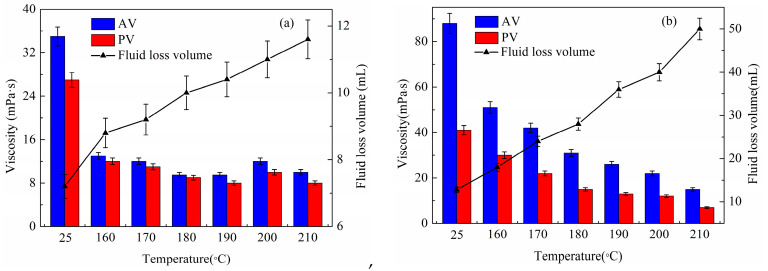
Effects of the filtrate reducer on the rheology and filtration performance of mud samples after aging at different temperatures: LMF (**a**) and Driscal Temp (**b**).

Figure 5a shows the rheology and filtration of mud samples containing LMF after aging at different temperatures. The results show that viscosity is stable, but filtration increases with the increase in aging temperature. Further, after aging at 210 °C, the filtration was only 11.6 mL. In contrast, the addition of Driscal Temp, a thickening filtrate reducer, increased both the viscosity and filtration before and after aging, reaching 50 mL after aging at 210 °C. Therefore, Driscal Temp has a greater viscosity-increasing effect and poorer temperature resistance and filtrate-reducing effect than LMF. As the aging temperature increased, the viscosity of drilling fluid containing LMF changed less, while the viscosity of drilling fluid with Driscal Temp decreased significantly, indicating that LMF has better high-temperature stability. The impact of high temperature on drilling fluid is, on the one hand, that it can reduce the hydration degree of clay, and on the other hand, that high temperature can cause the decomposition and failure of the treatment agent. The interaction between the two aspects leads to a decrease in drilling fluid viscosity and an increase in filtration loss after high-temperature aging. Therefore, compared to large molecule Driscal Temp, which has a significant viscosity-increasing effect, under high-temperature conditions, the long chain of large molecules in Driscal Temp is more prone to fracture, while the influence of temperature on the small molecule fluid loss reducer LMF is smaller, which is manifested as the viscosity of the drilling fluid being less affected by aging temperature. The tests confirm that LMF has minimal impact on rheology and good temperature resistance up to 210 °C.

The comparison results indicate that the performance of Driscal Temps is greatly affected by temperature, and as the temperature increases, the macromolecular chains of Driscal Temps gradually break and decompose, leading to a decrease in performance. Additionally, the performance decreases significantly after 170 °C, indicating that in water-based drilling fluid environments, 170 °C is the temperature resistance turning point of the filtrate reducer Driscal Temp. As the temperature increases, the performance of LMF is actually very stable, indicating that the temperature resistance performance of LMF is better than that of Driscal Temp. The main chain of LMF’s small molecule chain is a carbon–carbon bond, which has a large bond energy that is not easily broken and is not easily degraded at high temperatures. At the same time, LMF can also firmly adsorb on the surface of clay particles under hydrogen bonding, maintaining the hydration degree of clay particles and the colloidal stability of the drilling fluid.

### 2.6. Evaluation of Salinity Tolerance

**Figure 6:**
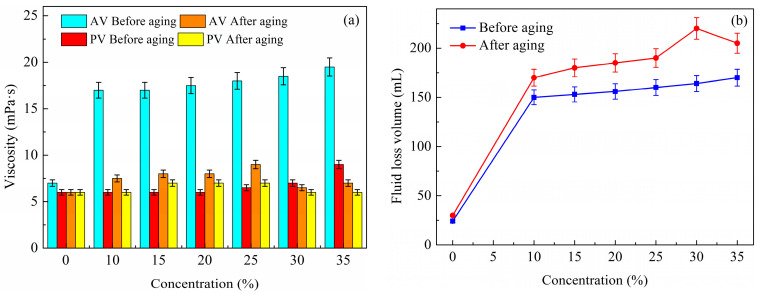

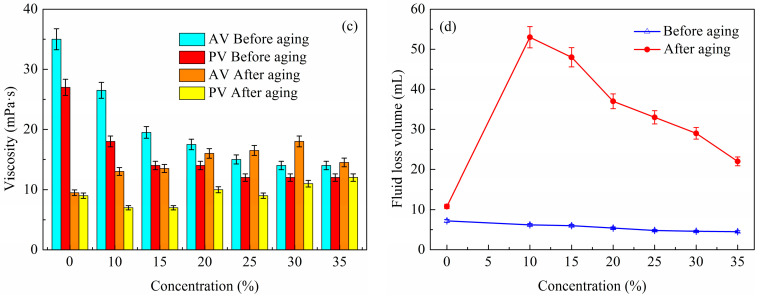
Rheology (**a**) and filtration performance (**b**) of mud samples before and after aging with different salt dosages, and effects of LMF on the rheology (**c**) and filtration performance (**d**) of mud samples before and after aging with different salt dosages.

As the salt content increases, the apparent viscosity and plastic viscosity of the base mud gradually increase. This is because the presence of salt compresses the diffusion hydration layer of the clay, causing some clay particles to aggregate [15,31]. The viscosity of the drilling fluid is influenced by the internal friction force between the solid phase (mainly clay particles) of the drilling fluid. Therefore, the agglomeration of clay particles under the influence of salt leads to an increase in internal friction between clay particles, resulting in an increase in viscosity. Figure 6c shows the rheology and filtration of mud samples containing 2 wt% LMF and different NaCl dosages. The results show that viscosity decreases before aging and increases after aging, although with only small variations, confirming that LMF has a minimal impact on the rheology. Further, the filtration increases with the increase in NaCl dosage for blank mud samples (Figure 6b), reaching over 150 mL at 10 wt% NaCl. For mud samples with LMF, filtration decreases with the increase in NaCl dosage and stays below 10 mL before and after aging. This is due to the small molecular weight of LMF, which has a higher adsorption capacity on the surface of clay compared to large molecule filtration reducers. So, under high salt conditions, clay particles protected by LMF can maintain a good degree of hydration. Under the action of LMF, the content of fine clay particles in the drilling fluid can be maintained, thereby significantly reducing the filtration of the drilling fluid. This indicates that LMF can greatly reduce filtration and has a high salinity tolerance.

As the salt content increases, the hydration layer of clay particles in the base slurry is continuously compressed, causing compression between clay particles, increasing internal friction between them, and increasing viscosity. However, the spatial grid structure is further compressed and damaged, resulting in an increase in filtration loss. After adding LMF, it adsorbs the surface of clay particles, enhancing the thickness of the hydration layer on the clay surface and hindering the aggregation between clay particles. However, as the salt concentration increases, some clay particles still aggregate into large particles, resulting in a wider distribution of clay particle size and instead forming smaller and denser mud cakes, resulting in a decrease in drilling fluid filtration. Overall, under different salt contents, LMF has a good filtration reduction effect because LMF molecular chains contain a large number of sulfonic acid groups. The sulfonic acid groups have good temperature and salt resistance and are not sensitive to salt. They can form stable conjugated systems with hydrophilic groups, such as hydroxyl groups on clay, preventing the invasion of salt ions and thereby improving overall temperature and salt resistance. At the same time, the molecular chain contains amide groups with strong adsorption, which are not easily affected by salt invasion and desorption, ensuring the effectiveness of the filter loss agent under high temperatures and high salt conditions.

### 2.7. Investigation of the Mechanism of Action of LMF

The addition of more LMF increased the absolute zeta potential of the particles in the drilling fluid before and after aging. Notably, the zeta potential of the mud samples generally remained constant with a 2 wt% LMF dosage, most likely because the polymer chains of LMF were adsorbed by the bentonite particles, which increases the thickness of the hydration shell and the electrostatic repulsion between the particles, thus enhancing the absolute zeta potential. Due to the presence of hydroxyl groups on the surface of clay particles, they are negatively charged, and the Zeta potential of the drilling fluid is usually negative. Zeta potential can reflect the stability of the drilling fluid system [18,32]. The more stable the drilling fluid dispersion system is, the higher the degree of hydration of clay particles and the greater the absolute value of Zeta potential. It is generally believed that when the absolute value of the Zeta potential of the drilling fluid system is greater than 35 mV, the drilling fluid system is relatively stable. Before and after aging, the absolute Zeta potential values of drilling fluid containing filter loss reducing agent LMF are all greater than 35 mV, indicating good stability of the drilling fluid system and LMF having a good adhesive protection effect. Therefore, the proposed filtrate reducer stabilizes the clay particles in the system by increasing the degree of hydration (preventing coalescence) and, thus, reducing the drilling fluid filtration.

**Figure 7:**
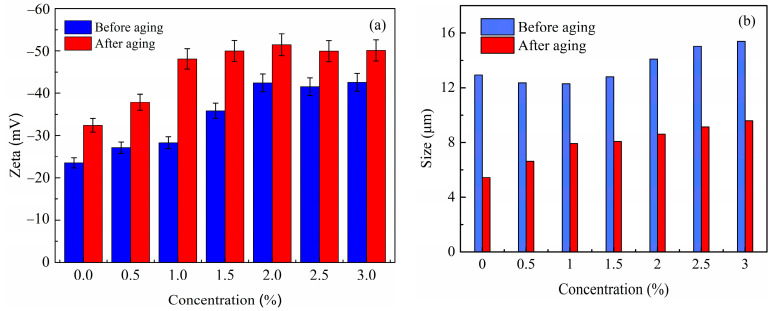
Effects of different LMF dosages on the zeta potential (**a**) and particle size (**b**) of mud samples before and after aging.

**Figure 8:**
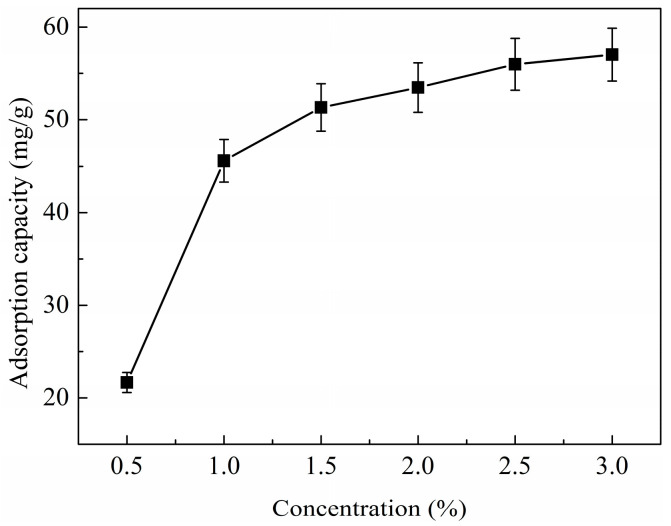
Effects of different LMF dosages on LMF adsorption on the bentonite surface.

The particle size of the mud samples also generally increased with the increase in LMF dosage (Figure 7b). This is attributed to the increased thickness of the hydration shell around the clay particles as a result of LMF adsorption. The adsorption of LMF on the clay particle surface also increased with the increase in LMF dosage (Figure 8). Further, when the LMF dosage reached 1.5 wt%, the curve of LMF adsorption on the clay particle surface plateaued, indicating adsorption saturation. In summary, LMF can adsorb a large amount on the surface of clay particles, and under high temperature and high salt conditions, it can also effectively adsorb on the surface of clay particles due to its small molecule characteristics and increases the hydration shell thickness, which increases the Zeta potential of the clay particles and inhibits particle aggregation and flocculation. During filtration, thin and dense mud cakes are formed, and thus, LMF exhibits its filtrate-reducing effect.

**Figure 9:**
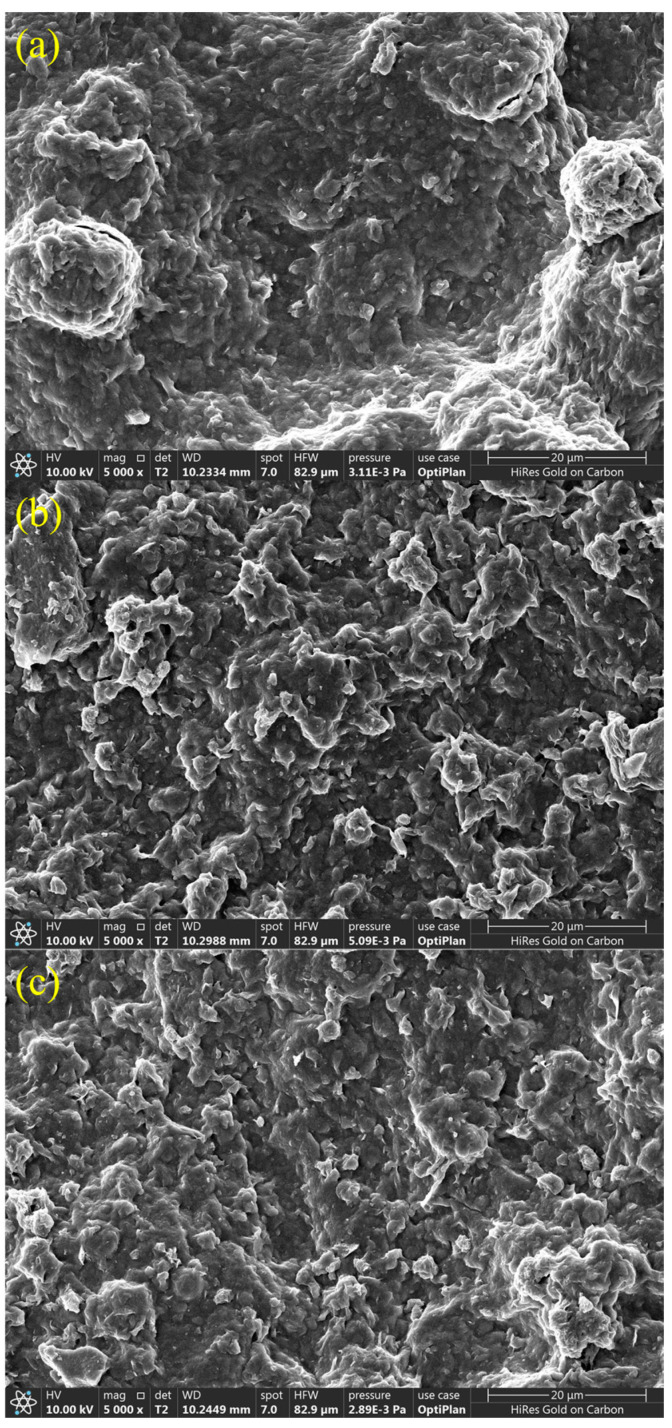
SEM images of filter cakes (×5000). Filter cake from blank mud sample (**a**), filter cake from drilling fluid with 2 wt% LMF (**b**), and filter cake from salt-saturated drilling fluid with 2 wt% LMF (**c**).

Figure 9 shows the SEM images of the filter cakes from the blank mud sample, drilling fluid with 2 wt% LMF, and salt-saturated drilling fluid with 2 wt% LMF after aging at 180 °C. The filter cake from the blank mud sample has a lumpy surface with large pores, and obvious large particles of clay can be seen, resulting in a high filtration amount (Figure 9a). In contrast, there are no large clay particles on the surface of the filter cake from the drilling fluid with 2 wt% LMF. This suggests that LMF adsorbs effectively on the clay particle surface and increases the hydration shell thickness, thus preventing severe clay particle aggregation at high temperatures. In the salt-saturated case, the sample shows clumps of clay particles and salt crystals, and microcracks can also be seen on the filter cake surface (Figure 9c). However, compared to the filter cake from the blank mud sample, the filter cake from the salt-saturated drilling fluid with 2 wt% LMF has no large pores or aggregated clay particles on its surface, which further confirms the significant filtrate-reducing effect of LMF at high temperature and high salinity.

## 3. Conclusions

In this work, we developed a low-molecular-weight filtrate reducer with high-temperature resistance, LMF, using free radical polymerization. LMF exhibits a weight-average molecular weight of 3819 and a starting temperature for thermal decomposition of 300.7 °C, indicating good thermal stability. Further, LMF can effectively improve the viscosity of drilling fluids and reduce their filtration. The filtration of drilling fluid containing 2 wt% LMF was 7.2 mL, a reduction of 70% compared with that of the blank drilling fluid. Further, after aging at 210 °C for 16 h, the filtration of drilling fluid containing 2 wt% LMF was 11.6 mL. Crucially, after the addition of 35 wt% NaCl and aging at 180 °C for 16 h, the filtration of drilling fluids containing 2 wt% LMF was only 22 mL. In addition, we found that LMF is effectively adsorbed on the surface of clay particles and increases the thickness of their surrounding hydration shells, thereby enhancing the zeta potentials of the particles and preventing their aggregation and flocculation. This results in the formation of thin and dense filter cakes during filtration, which reduces drilling fluid filtration. Overall, this work can promote the application of small molecular weight polymers in the filtration reduction in high-temperature water-based drilling fluids.

## 4. Materials and Methods

### 4.1. Materials

Acrylamide (AM, AR) and 2-acrylamido-2-methyl-1-propanesulfonic acid (AMPS, AR) were purchased from Shanghai Macklin Biochemical Technology Co., Ltd. (Shanghai, China). Sodium hydroxide (NaOH, AR), ammonium persulfate (APS, AR), and sodium chloride (NaCl, AR) were purchased from Sinopharm Chemical Reagent Co., Ltd. (Shanghai, China). Tertiary dodecyl mercaptan (TDDM, AR) was purchased from Aladdin Biochemical Technology Co., Ltd. (Shanghai, China).

### 4.2. Preparation of LMF

**Scheme 1:**
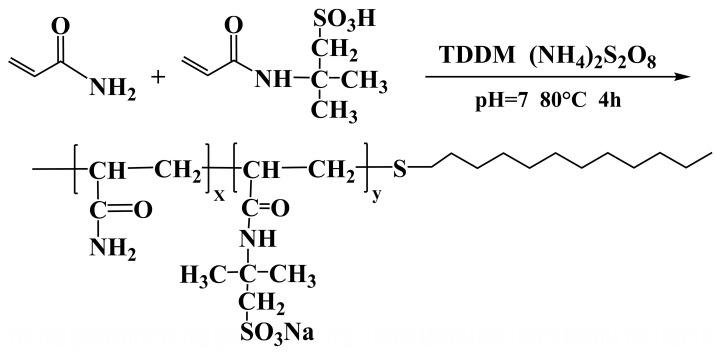
The synthesis procedure and structural formula of LMF.

AM, AMPS, and TDDM were weighed and added to deionized water and then stirred until dissolved. The obtained solution was adjusted with 30 wt% NaOH solution until pH = 7. The solution was then transferred into a three-neck round-bottom flask equipped with a stirring device. The flask was heated in a water bath at 80 °C under nitrogen protection and stirred at a speed of 350 rpm. Once the temperature of the reaction mixture reached the reaction temperature, aqueous APS (0.5 mL) was added to initiate the reaction. The reaction was continued for 4 h with heating and stirring. The product was then dried and milled, yielding LMF. The LMF product was repeatedly centrifuged and washed with deionized water and ethanol, and the washed sample was then dried and milled for characterization. The synthesis process and possible chemical structure of LMF are shown in Scheme 1.

### 4.3. Characterization

#### 4.3.1. FTIR Measurements

LMF and KBr were compressed to fabricate semi-transparent sheets. The sample sheets were placed in a Fourier transform infrared spectrometer (IRTRacer-100, Shimadzu, Kyoto, Japan), and the FTIR spectrum of LMF was measured between 4000 and 400 cm^−1^. The resolution was 4 cm^−1^, and the number of scans was 32.

#### 4.3.2. TGA Measurements

After LMF (6 mg) had been weighed in a crucible, it was placed in the TGA device (TGA2, Mettler Toledo, Zurich, Switzerland). Measurements were carried out in a nitrogen atmosphere within the temperature range from 40 to 600 °C at a heating rate of 5 °C/min.

#### 4.3.3. GPC Measurements

The molecular weight of LMF was determined using a differential refractive index detector (WATERS 1515-2414, Waters, Milford, MA, USA). Tetrahydrofuran was used as the solvent during measurement at a mobile phase flow rate of 1.0 mL·min^−1^ at 40 °C.

### 4.4. Evaluation of Rheology and Filtration Performance

#### Preparation of Mud Samples

Distilled water (400 mL) was added to a mud cup, and anhydrous sodium carbonate (0.69 g) and bentonite (20.0 g) were successively added while the mixture was continuously stirred for 20 min. The sample was then sealed and cured at room temperature for 24 h to form the mud samples for evaluation.

LMF was then dosed into mud samples in various amounts and stirred for 20 min. The rheological properties of the dosed mud samples were measured using a six-speed rotational viscometer (ZNN-D6, Qingdao Tongchun, Qingdao, China). The filtration of the mud samples was measured using an API filter press (SD6A, Qingdao Tongchun, China). In addition, mud samples with different LMF dosages were added to an aging tank and aged at 180 °C for 16 h in a roller oven. The aged mud samples were transferred back to the mud cup to measure their rheology and filtration performance again after stirring for 20 min. The rheological properties of drilling fluid are calculated using the following formula [15]:

(1) $$\mathrm{Apparent}\;\mathrm{Viscosity}\;(\mathrm{AV})=\frac{\theta_{600}}{2}\left(\mathrm{mPa}\cdot s\right)$$

(2) $$\mathrm{Plastic}\;\mathrm{Viscosity}\;(\mathrm{PV})={\;\theta }_{600}-\theta_{300}\;(\mathrm{mPa}\cdot s)$$

where θ_600_ and θ_300_ are the values of the rotary viscometer at the corresponding speed, respectively.

### 4.5. Evaluation of Temperature Resistance

Mud samples were dosed with 2 wt% LMF and stirred for 20 min. The samples were then aged at different temperatures for 16 h to measure the rheology and filtration performance of mud samples with different aging temperatures.

### 4.6. Evaluation of Salinity Tolerance

Mud samples were dosed with 2 wt% LMF and various amounts of NaCl to measure their rheology and filtration performance before and after aging at 180 °C.

### 4.7. Investigation of the Mechanism of Action of LMF

Mud samples were dosed with different amounts of LMF, and the zeta potentials and particle sizes of the samples before and after aging were measured using a laser nanoparticle size analyzer (Zetasizer Nano ZS90, Malvern, UK) and a laser particle size analyzer (Mastersizer3000, Malvern, UK). The microscopic morphology of the filter cakes from a blank mud sample, drilling fluid with 2 wt% LMF, and salt-saturated drilling fluid with 2 wt% LMF were observed using SEM (Nova NanoSEM 450, FEI Company, Hillsboro, OR, USA).

The filtrate reducers exert a filtration-reducing effect by adsorbing on the surface of clay particles. The amount of adsorption of filtrate reducers on the surface of the clay is the key factor affecting its filter loss performance. Total organic carbon analysis of LMF adsorbed on the surface of clay for samples with different LMF dosages (TOC-L, Shimadzu, Japan) and LMF adsorbed on the surface of bentonite for samples with different LMF dosages (TOC-LCPH/CPN, Shimadzu, Japan) was carried out. The TOC values of 30 mg untreated bentonite and bentonite treated with different LMF dosages were measured after heating to 900 °C. The LMF-treated bentonite samples were acquired by centrifuging 50 mL of drilling fluid containing different LMF dosages. Before measurement, each material was ground to 200 mesh. The amount of adsorbed LMF was calculated using Equation (3) [9].

(3) $$M_{\varphi }=\frac{M_{3}\;-\;M_{1}}{M_{2}}\;\times 1000$$

Here, M_φ_ is the amount of adsorbed LMF, and M_1_, M_2_, and M_3_ are the TOCs of 30 mg untreated bentonite, LMF, and LMF-treated bentonite, respectively.

## Figures and Tables

**Table 1:** LMF relative molecular weights.

Weight-Average Molecular Weight (*M*_w_)	Number-Average Molecular Weight (*M*_n_)	Z-Average Molecular Weight (*M*_z_)	Peak Molecular Weight (*M*_p_)	Z-Average Molecular Weight (*M*_z+1_)	Dispersion Index (*D = M*_w_/*M*_n_)
3819	2507	6726	2640	11,306	1.761093

## Data Availability

Not applicable.
